# Evidence for an Ordering Transition near 120 K in an Intrinsically Disordered Protein, Casein

**DOI:** 10.3390/molecules26195971

**Published:** 2021-10-01

**Authors:** Natalya A. Maslennikova, Elena A. Golysheva, Sergei A. Dzuba

**Affiliations:** 1 Department of Physics, Novosibirsk State University, Novosibirsk 630090, Russia; Natalya.A.Maslennikova@gmail.com; 2 Voevodsky Institute of Chemical Kinetics and Combustion, Russian Academy of Sciences, Novosibirsk 630090, Russia; elenabiochem@gmail.com

**Keywords:** EPR, electron spin echo, spin labels, stochastic molecular librations, lipid bilayers, molecular packing

## Abstract

Intrinsically disordered proteins (IDPs) are proteins that possess large unstructured regions. Their importance is increasingly recognized in biology but their characterization remains a challenging task. We employed field swept Electron Spin Echoes in pulsed EPR to investigate low-temperature stochastic molecular librations in a spin-labeled IDP, casein (the main protein of milk). For comparison, a spin-labeled globular protein, hen egg white lysozyme, is also investigated. For casein these motions were found to start at 100 K while for lysozyme only above 130 K, which was ascribed to a denser and more ordered molecular packing in lysozyme. However, above 120 K, the motions in casein were found to depend on temperature much slower than those in lysozyme. This abrupt change in casein was assigned to an ordering transition in which peptide residues rearrange making the molecular packing more rigid and/or more cohesive. The found features of molecular motions in these two proteins turned out to be very similar to those known for gel-phase lipid bilayers composed of conformationally ordered and conformationally disordered lipids. This analogy with a simpler molecular system may appear helpful for elucidation properties of molecular packing in IDPs.

## 1. Introduction

The protein functions are determined by their three-dimensional tertiary structures. However, there exist proteins possessing unstructured regions of significant size, being nevertheless biologically active; these proteins are called intrinsically disordered proteins (IDPs), as reviewed in [1,2,3,4,5,6,7]. IDPs carry out various functional roles, such as signal transduction and storage of small molecules. IDPs recognize proteins, nucleic acids, and other types of binding molecules and accelerate interactions and biochemical reactions between bound partners; biological activities of IDPs complement those of structured proteins.

Characterization of IDPs and intrinsically disordered regions (IDRs) in proteins can be done by different spectroscopic methods: nuclear magnetic resonance (NMR), small-angle X-ray scattering (SAXS), single-molecule fluorescence, mass spectrometry—for a recent review see [8]. Among others, electron paramagnetic resonance (EPR) spectroscopy has proven to be a valuable tool to study functional properties of IDPs [9,10,11,12].

An intriguing property of many IDPs is their ability to undergo disorder-to-order transitions upon target binding, when specific recognition protein residues adopt secondary or tertiary structural elements upon interaction with a binding partner [1,2,3,4,5,6,7,8,9]. These transitions contribute to protein function by facilitating the binding process and accelerating biochemical reactions.

In this work we report observations evidencing that disorder-to-order transitions may be induced also by temperature increase, at cryogenic temperatures. We employ pulse EPR based on electron spin echo (ESE) phenomenon, within an approach allowing for spin-labeled molecules detection of their stochastic molecular librations (stochastic small-angle oscillations of a molecule as a whole) [13,14,15]. These librations are a general property of molecular solids and highly viscous liquids. ESE-detected librations in molecular systems are determined by the nearest surrounding of a molecule [16,17]. Therefore, this approach may serve as a tool to study molecular packing in the molecular systems.

Using this approach, molecular packing was studied in gel-phase biological membranes (lipid bilayers) [16,17,18,19] and in glassy ionic liquids [20,21,22,23]. Further, interaction of free fatty acids with proteins [24] and interaction of sugars with biological membranes [25] was investigated. Stochastic molecular librations are sensitive to a so-called dynamical transition in proteins and in other biological systems [16,19,26,27,28], which is known occur between 170 and 220 K from numerous neutron-scattering experiments and MD simulations to—see e.g., [29,30,31] for recent references. Note however that the nature of this transition is still heavily debated.

As a model IDP we employ here bovine casein. Caseins are the main proteins of milk and belong to a family of phosphorylated proteins including four genetic types (αS1-, αS2-, β- and κ-caseins); all these proteins are considered as IDPs [32,33]. We study here as a reference also a globular enzyme, a hen egg white lysozyme, which possesses a well-documented conformational stability [34]. As a spin label we used 4-(2-iodoacetamido)-TEMPO (IASL). Additionally, we use nitroxide 4-hydroxy-2,2,6,6-tetramethylpiperidine-*N*-oxyl (TEMPOL) as a spin probe. Spin labeling of proteins was performed within the protocols described for casein in [12] and for lysozyme in [27]. The results of the present study have shown that labels are located at the protein-water interface of the hydrated proteins.

The hydration level *h* used in this work was 0.4 g of water per gram of casein (*h* = 0.4). For this hydration level, proteins are known to be enveloped with a water monolayer covering its surface to maintain its activity [35]; this hydration level is often employed in protein studies by neutron scattering, see e.g., [36,37]. For comparison purpose, we investigated here both hydrated and dry protein samples.

## 2. Results

**Figure 1:**
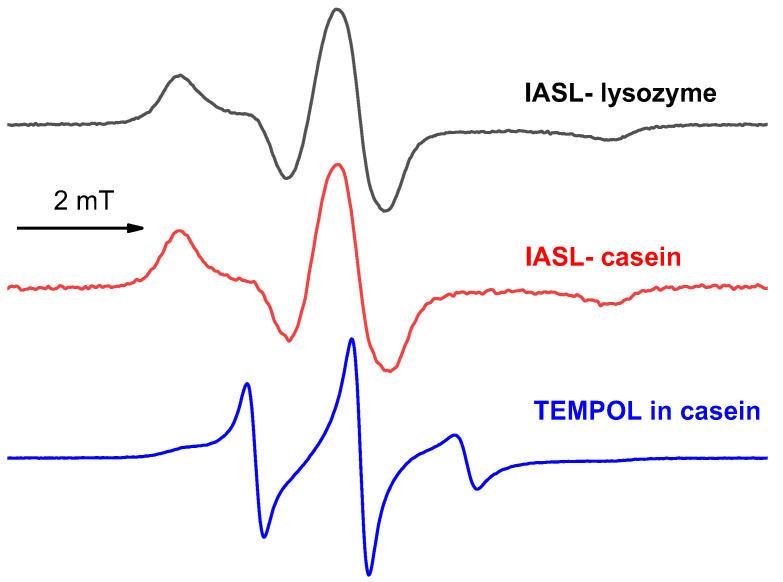
CW EPR spectra at room temperature for D_2_O-hydrated spin-labeled lysozyme and casein and for TEMPOL in hydrated casein. The hydration level *h* = 0.4, the nitroxide/protein molar ratio is near 1/100 in all cases.

Conventional continuous wave (CW) EPR spectra of the hydrated samples recorded at room temperature are shown in Figure 1. For IASL-labeled proteins, these spectra are typical for immobilized nitroxides, which evidences covalent binding of the spin labels to the protein. For TEMPOL in hydrated casein, the CW EPR spectrum shows a motional narrowing that is expected because of its hydrophilic nature and the location therefore in the hydration layer (a small admixture of the broad wings seen in Figure 1 implies that TEMPOL partially is adsorbed on the protein surface). In all cases, CW EPR spectra in Figure 1 look similar to those known for diluted conditions [38] (i.e., the spectra are not broadened by inter-spin magnetic interactions, dipole-dipole or exchange), which evidences that spin labels and TEMPOL are diluted in their surroundings.

Additional information on the spin label location in proteins can be obtained in three-pulse stimulated electron spin echo envelope modulation (ESEEM) experiments on D_2_O-hydrated biological systems [39,40,41]. Figure S1 in Supplementary Materials shows results of these measurements, with the original ESEEM data treated as described in [39,40,41]. The ESEEM effect is induced by hyperfine interaction of the electron spin with the nearest deuterium nuclei; the amplitude of the effect reflects the distance of the spin label to the hydration layer [39,41]. From comparison with literature data for D_2_O-hydrated lipid bilayers [40] it was concluded that spin labels are located on the peptide surface, directly exposing to the water.

EPR spectra of nitroxide spin labels are determined by the nitrogen spin projection onto the direction of the magnetic field and, at low temperatures, by anisotropy of the *g*-factor and hyperfine interactions [38]. The anisotropy of magnetic interactions results in anisotropic spectral broadening that is seen in Figure 1. In ESE experiments, normally the microwave pulses excite only a small portion of the spectrum. This selective excitation at spectral positions of different degree of anisotropy allows studying the anisotropy of spin relaxation. Low-amplitude orientational molecular motions (librations) result in anisotropic relaxation manifesting itself in a two-pulse ESE experiment as a different relaxation rate for different spectral positions: faster relaxation is observed for the spectral position with the larger spectral anisotropy.

**Figure 2:**
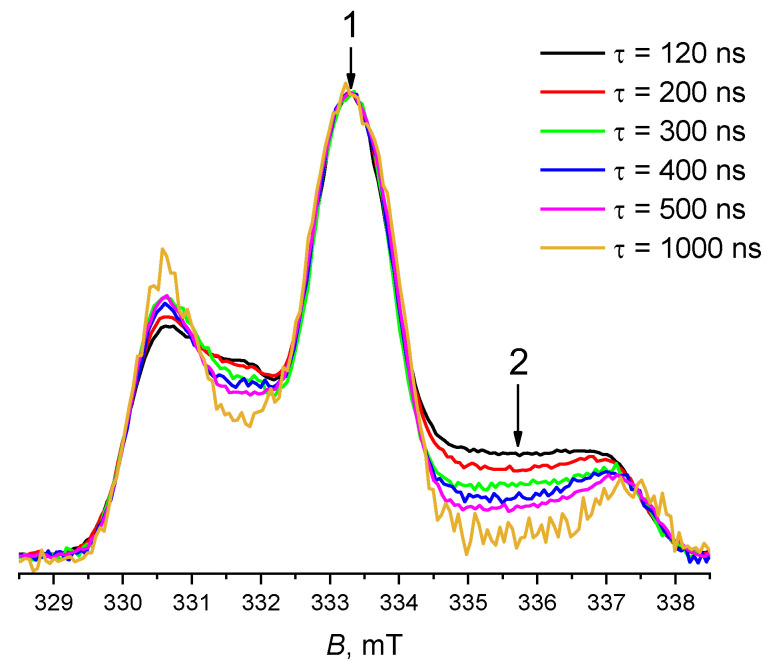
Echo-detected EPR spectra for hydrated IASL-lysozyme (the hydration level *h* = 0.4) at 178 K measured with τ fixed at 120, 200, 300, 400, 500 and 1000 ns. Spectra are normalized by their maximal intensity. The spectral positions 1 and 2 are indicated with the smallest and largest anisotropies, respectively.

That is just what is seen in Figure 2 which shows echo-detected EPR spectra for hydrated IASL-lysozyme measured at 178 K. The data were obtained with different time delays τ and normalized to the maximum of the central peak (spectral position 1). Such normalization is convenient for excluding field-independent spin-relaxation mechanisms and presenting the anisotropic nature of spin-relaxation. One can see that the echo signal decays with τ increase more rapidly for the high-field hyperfine component, which is the most anisotropic. This effect evidences that the observed field-dependent relaxation is induced by orientational motion. Then, Figure 2 shows that relaxation at the middle of the high-field component (spectral position 2) is faster than that at the two outer shoulders of the spectrum. As the shoulders refer to the so-called canonical orientation of the magnetic tensor to the external magnetic field, being therefore less sensitive to reorientations, this behavior is typical for spin-relaxation induced by low-amplitude molecular librations [15,16,17]. Note also that, as echo decays are only due to relaxation processes in the spin system, the motion is to be stochastic.

The effects seen in Figure 2 strongly depends on temperature, which is illustrated in Figure S2 of Supplementary Materials.

Note that ESE signal decay, except of molecular motion of the spin-labeled molecules, is induced in solids also by other physical mechanisms—spectral diffusion due to spin relaxation of nearby nuclear and electron spins, spin-spin interaction between spins, spin-lattice relaxation of the spins under consideration—see [42,43,44,45] for more details. Fortunately, some of these additional mechanisms normally are too slow (spin-lattice relaxation) to influence the two-pulse echo decay; the others are mostly field-independent (spin relaxation of the nearby spins) and so may be excluded by comparison echo decays taken at two field possessing different spectral anisotropy. The only exception is so-called “instantaneous diffusion” mechanism of spectral diffusion in ESE—see e.g., [14]. However, this mechanism may be assumed to temperature-independent, so its contribution may be selected at low temperature and then simply subtracted from data at other temperatures.

The pure contribution of librational motion may be obtained at X-band EPR from the ratio of ESE decays at two field positions shown in Figure 2 which is expected to depend exponentially on the time delay τ (Equation (1)) [15,16,17]:

(1) $$E_{2}(2\tau )/E_{1}(2\tau )=const\;\exp(-2\tau \Delta W)$$

where Δ*W* may be termed as an anisotropic relaxation rate. The representative examples of the decays *E*_1_(2τ) and *E*_2_(2τ) and their ratios are shown in Figure S3 of Supplementary Materials, which indeed demonstrates exponential decay.

**Figure 3:**
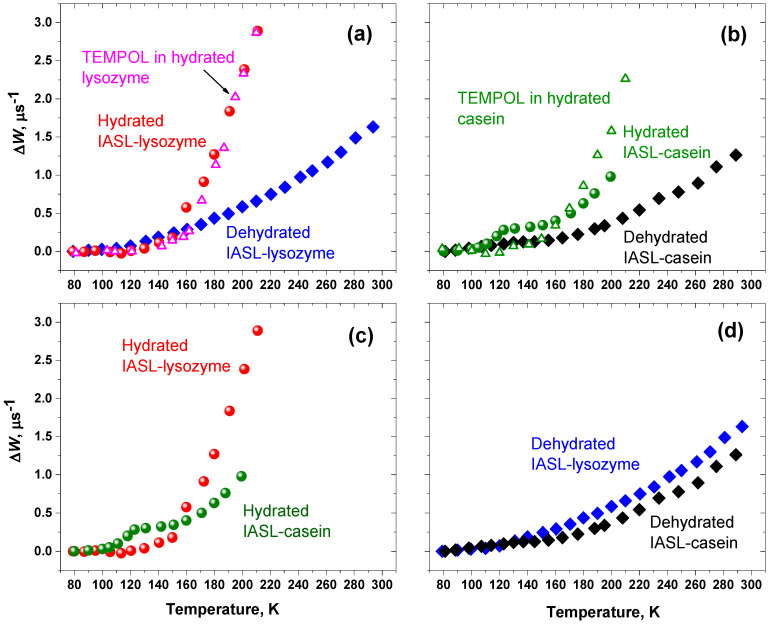
Temperature dependencies of the anisotropic relaxation rate Δ*W*: (**a**) Dehydrated and hydrated IASL-lysozyme and TEMPOL in hydrated lysozyme; (**b**) Dehydrated and hydrated IASL-casein and TEMPOL in hydrated casein; (**c**) Hydrated IASL-lysozyme and hydrated IASL-casein; (**d**) dehydrated IASL-lysozyme and dehydrated IASL-casein. The experimental uncertainty is close to the size of the symbols. Data for TEMPOL in hydrated lysozyme are taken from [27].

The Δ*W* data, obtained in this way (with subtracting the data at 79 K), are presented in Figure 3 as functions of temperature. For comparative purposes, data in Figure 3 are grouped in pairs: dehydrated and hydrated proteins (Figure 3a,b), hydrated lysozyme and hydrated casein (Figure 3c), and dehydrated proteins (Figure 3d).

Data in Figure 3 were obtained mostly upon the temperature increase. At some selected temperatures, measurements were also taken upon temperature decrease; the results demonstrated a good reproducibility.

Low-temperature librations manifest also themselves in conventional CW EPR spectroscopy [46,47,48,49,50]: these motions reduce the total spectral splitting of nitroxide EPR spectra; except of stochastic librations, dynamical librations contribute to this reduction as well. This splitting with a good accuracy presents the partly motion-averaged doubled principal value $A_{zz}$ ≡ $\langle A_{\left|\right|}\rangle$ of the hyperfine structure tensor, 2$\langle A_{\left|\right|}\rangle$, where angular brackets imply the averaging. In the model of uniaxial librations around an axis lying in the NO plane, the $\langle A_{\left|\right|}\rangle$ is determined by the relation (Equation (2)) [46]

(2) $$\langle A_{\left|\right|}\rangle \approx A_{\left|\right|}^{0}-\left(A_{\left|\right|}^{0}-A_{⊥}^{0}\right)\langle \alpha^{2}\rangle$$

where $A_{⊥}$ is the principal value for the perpendicular orientation of the tensor in the approximation of its axial symmetry. The superscript ‘0’ denotes that $A_{\left|\right|}$ and $A_{⊥}$ values are taken at *T* = 0 K. CW EPR spectra for different temperatures for hydrated spin-labeled lysozyme and casein are shown in the Supplementary Materials Figure S4, temperature dependence of the $\langle A_{\left|\right|}\rangle$ value is presented in Figure S5. The $A_{\left|\right|}^{0}$ values were obtained by linear extrapolation of the $\langle A_{\left|\right|}\rangle$ values to zero temperature, and $A_{⊥}^{0}$ value was taken equal to 0.8 mT [51].

**Figure 4:**
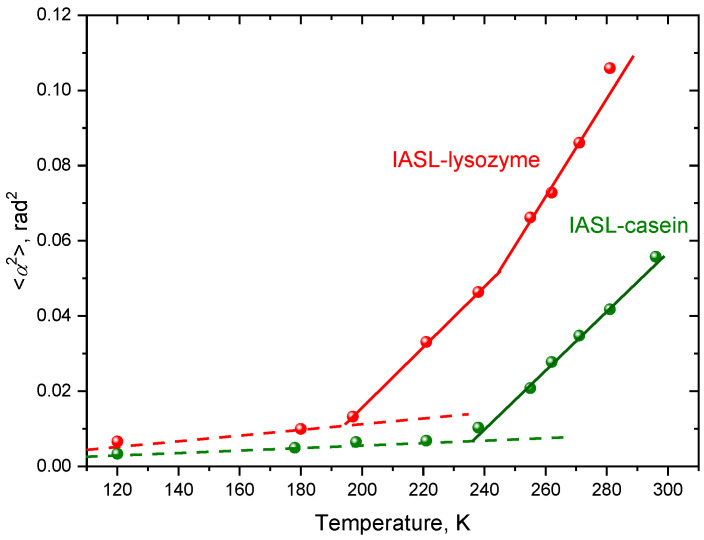
Temperature dependencies of the $\langle \alpha^{2}\rangle$ mean squared angular amplitudes of the librations derived from CW EPR spectra (see Figures S4 and S5) for hydrated casein and lysozyme. Dashed lines show low-temperature linear dependencies predicted for harmonic librations; solid lines are drawn to guide the eye.

Data treatment of the $\langle A_{zz}\rangle$ values using Equation (2) allows us to obtain $\langle \alpha^{2}\rangle$ values; the results are presented in Figure 4. One can see that below 195 K for lysozyme and below 235 K for casein the $\langle \alpha^{2}\rangle$ temperature dependencies may be approximated with straight lines, which corresponds to harmonic vibrations of atoms in solids [46]. At higher temperatures a noticeable deviation from the linear dependencies is observed. The deviation occurs at 195 ± 5 K for lysozyme and at 235 ± 5 K in casein; it may be related with a transition from harmonic to anharmonic vibrations [29,30].

## 3. Discussion

Data in Figure 3a,b show that for both spin-labeled proteins for their hydrated states, the Δ*W* value increases faster than that for the dehydrated ones. This means that the matrices of the hydrated proteins are more mobile in comparison with the dehydrated samples. And Figure 3d demonstrates closeness of temperature dependencies for both dehydrated proteins which may imply proximity of their intermolecular packings in this state.

The Δ*W* temperature dependencies for TEMPOL are compared in Figure 3a,b with the analogous data for spin-labeled proteins. One can see that for lysozyme (Figure 3a) the two dependencies are close to each other while for casein (Figure 3b) the dependencies are remarkably different: the increase of Δ*W* for TEMPOL starts at a noticeably higher temperature, and then its temperature dependence becomes much steeper. These features imply that for lysozyme the molecular motions in the hydration layer and for the protein backbone (to which the spin label is assumed to be attached) are coupled while in casein these two motions occur more or less independently, with motions of protein backbone being relatively restricted.

The main result of this work is presented in Figure 3c which shows that the Δ*W* temperature dependencies for the two proteins differ significantly. First, molecular mobility in hydrated casein appears at 100 K, while in hydrated lysozyme—only at 130 K. This certainly implies that in casein there is more freedom available for molecular reorientations. Second, for casein a kink in the temperature dependence near 120 K is seen, above which the dependence becomes remarkably slower.

As stochastic molecular librations are governed by the surrounding of the molecule (see [16,17,27] and references therein), this kink may indicate a transition in the intermolecular packing. Such a transition with the slowing temperature dependence may appear if the freedom available in the IDP structure promotes the protein repacking, to make its structure more cohesive and/or more rigid, even in comparison with the globular protein. In the dehydrated state (see Figure 3d) two proteins behave very similarly which evidences that the observed features are an intrinsic property of the hydrated state, i.e., when proteins possess their functionality.

It is worth noting in this respect that the disorder-to-order transition in IDPs is widely recognized as a common property of IDPs upon their interaction with a binding partner [1,2,3,4,5,6,7,8,9,34]. Data of neutron and light scattering techniques [37] show that β-casein partially folds and stiffens upon calcium binding, and that in the unfolded state it is softer than folded proteins—the features analogous to those observed here, with the difference that the transition here is induced by the temperature increase.

Low-temperature ESE-detected stochastic molecular librations found in other globular proteins demonstrated the similar behavior with those observed here for lysozyme. For human serum albumin [24], Na, K-ATPase [50] and haemoglobin [48] the rapid increase of the Δ*W* value (denoted in these papers in a somewhat different way) was found for the temperature interval of 160–180 K which is consistent with data in Figure 3a. Protein dynamics studied by variable temperature NMR relaxation [52] revealed that IDP (C-terminus of the nucleoprotein of the Sendai virus, Sendai Ntail) possesses much lower energy of activation for motions of protons associated with the protein-solvent interface, as compared to globular proteins.

CW EPR data obtained in literature for the $\langle \alpha^{2}\rangle$ temperature dependencies in globular proteins hemoglobin [48,49], human serum albumin [24,49], β-lactoglobulin [49] look very similar to those shown here in Figure 4 for lysozyme: in all these cases the $\langle \alpha^{2}\rangle$ value starts to increase with temperature near 200 K. Quasielastic neutron scattering has showed for β-casein a remarkable increase of the mean-squared amplitude of atomic molecular motion, $\langle x^{2}\rangle$, at 100 K and then at 240 K [36]. For human IDP tau, $\langle x^{2}\rangle$ was found to increase at 100 K and then near 250 K [53].

To further support the suggested explanation of the kink for the Δ*W* temperature dependence for casein near 120 K as evidence for the disorder–order transition, we turn to comparison with the analogous ESE studies of stochastic molecular librations in spin-labeled gel-phase phospholipid bilayers composed either of conformationally ordered or conformationally disordered lipids. These studies were performed for the bilayers composed of saturated 1,2-dipalmitoyl-*sn*-glycero-3-phosphocholine (DPPC) lipids [16,19,28], mono-unsaturated 1-palmitoyl-2-oleoyl-*sn*-glycero-3-phosphocholine (POPC) lipids [16,28] doubly-unsaturated 1,2-dioleoyl-*sn*-glycero-3-phosphocholine (DOPC) lipids [17,28]. In these systems, DPPC presents an example of conformationally ordered molecule while POPC and DOPC are conformationally disordered. Therefore, one may hypothesize a similarity of intermolecular packing in lysozyme and in gel-phase DPPC bilayer, from one hand, and in casein and in gel-phase POPC (or DOPC) bilayer, from the other. Indeed, in the DPPC bilayer ESE-detected stochastic librations were found to start above 130 K while in the POPC bilayer they appear already at 100 K, possessing, however, lower activation energy as compared with DPPC [16].

We have to note here that these two starting temperatures, 130 and 100 K, seem to be quite a general property of ESE-detected stochastic molecular motions in biological systems [54,55]. Some evidence exists that at 130 K motions appear because of bending of the molecular structure while at 100 K the source of motions could be torsional oscillations [55]. In all the cases, stochastic molecular librations in biological systems develop because of cooperative effects [54,55].

The POPC molecule possesses a double bond in one of the acyl tails while DOPC molecule possesses two double bonds in two acyl chains. So, one may suggest that DOPC bilayer is more disordered than the POPC one. Unexpectedly, the Δ*W* temperature dependence for DOPC bilayer was found to occupy an intermediate position between DPPC and POPC bilayers [17]. This result was explained by the possibility for the DOPC molecule to rearrange its two tails beyond the double bonds in a parallel way, therefore adopting a more ordered structure. This disorder–order transition in the gel-phase DOPC bilayer starts near 130 K; it was found to be reversible with temperature increase or decrease [17].

**Figure 5:**
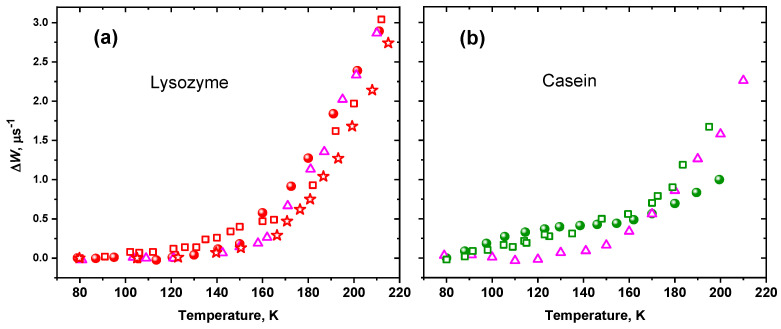
Temperature dependencies of anisotropic relaxation rate Δ*W* for hydrated proteins (the same data as in Figure 3) in comparison with literature data for gel-phase lipid bilayers: (**a**) IASL-lysozyme (circles), TEMPOL in lysozyme (triangles, data are taken from [27]), spin-labeled stearic acid (the label is at 5th carbon position of the acyl chain) in DPPC bilayer (squares, data are taken from [16]) and spin-labeled lipid heads in DPPC bilayer (stars, data are taken from [16]); (**b**) IASL-casein (circles), TEMPOL in casein (triangles) and spin-labeled at 5th carbon position stearic acid in DOPC bilayer (squares, data are taken from [17]).

In Figure 5 we compare the Δ*W* values found here for spin-labeled lysozyme and casein with those for spin-labeled DPPC [16] and DOPC [17] gel-phase bilayers. One can see a rather good agreement between the data for lysozyme and DPPC bilayer, from one hand, and between the data for casein and DOPC bilayer, from the other. So, rearrangement of the residues in the casein structure, making them more ordered, as it was suggested for the gel-phase DOPC bilayer, also may be employed as explanation of the kink in the observed Δ*W* temperature dependence for casein.

Regarding the CW EPR data, for the spin-labeled DPPC bilayer the $\langle \alpha^{2}\rangle$ value was found to start to increase above 200 K [19], which is also in agreement with data in Figure 4 for lysozyme. For spin-labeled DOPC bilayer, when the spin label is located near the bilayer surface, the $\langle \alpha^{2}\rangle$ value starts to increase at 240 K [28]. This result is consistent with that observed here in Figure 4 for casein. However, for the spin label in the interior of the DOPC bilayer [28] (the label is at the 16th carbon position of the acyl chain), the starting temperature of the $\langle \alpha^{2}\rangle$ increase is diminished down to 160 K, which implies that in the DOPC bilayer interior the motions become too intensive to be compared with those in casein.

Probably the starting temperature point for increasing the $\langle \alpha^{2}\rangle$ value that is derived from the CW EPR spectra—either ~200 or ~240 K for structured globular proteins and IDPs, respectively—could be a general property of both these protein types. To better understand the observed phenomena, further experimental and theoretical investigations are needed.

## 4. Materials and Methods

**Scheme 1:**
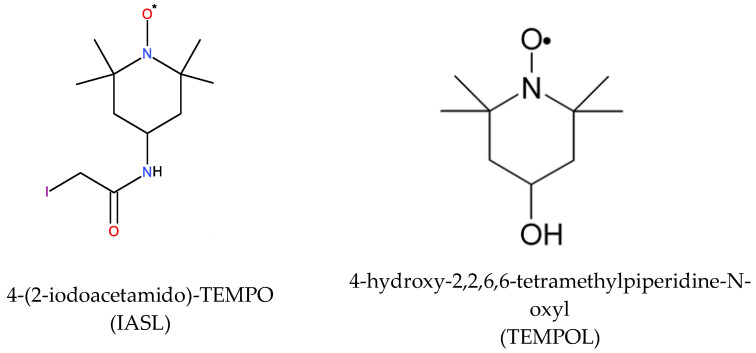
Chemical structures of nitroxide spin labels.

Casein from bovine milk was purchased from Sigma-Aldrich (Saint Louis, MO, USA), hen egg white lysozyme was from PanReac AppliChem (ITW reagents, Darmstadt, Germany). Both proteins were studied in the hydrated (water/protein mass ratio *h* = 0.4) and dehydrated states. As a spin label we used 4-(2-iodoacetamido)-TEMPO, kindly provided by I. A. Grigoryev and I. A. Kirilyuk, the chemical structure is given in Scheme 1. Additionally, nitroxide 4-hydroxy-2,2,6,6-tetramethylpiperidine-*N*-oxyl (TEMPOL, see Scheme 1) was used, purchased from Sigma-Aldrich (Saint Louis, MO, USA).

Casein spin labeling was performed using protocol described in [12]. Casein in the amount of 1.5 mg was dissolved in a buffer containing 4 M guanidinium chloride, 20 mM EDTA, 0.1 M Tris-HCl pH 6.0. Then a 1000-fold excess of tris(2-carboxyethyl)phosphine (TCEP) was added. The sample was stirred for 4 h at 55 °C, and TCEP was removed by centrifugation on a Amicon Ultra-15 concentrator with a 3 kDa cutoff (Millipore, Burlington, MA, USA). Then a 50-fold excess of spin label 4-(2-iodoacetamido)-TEMPO was added and the sample was stirred for 16 h at 50 °C. The excess of the spin label was removed by centrifugation.

Lysozyme spin labeling was performed as described in [27]. 7.5 mg of lysozyme was dissolved in 100 µL of 50 mM sodium acetate buffer (pH = 5). The spin label 4-(2-iodoacetamido)-TEMPO was taken at the amount of 6.3 mg and then dissolved in 20 µL of methanol. Then it was diluted 10-fold in sodium acetate buffer (making the total volume of 200 µL). This solution was added to the lysozyme solution and the mixture was stirred at 40 °C for 24 h. The excess of the spin label was removed by centrifugation at 14,000× *g* using a 10 kDa molecular mass cut-off filter (Amicon, Millipore, Bedford, MA, USA). In the previous studies [27] by MALDI-TOF mass spectroscopy (a Bruker Daltonics Ultraflex spectrometer, operating on a positive mode, Bruker, Rheinstetten, Germany) there was found that the most likely spin label is covalently attached to the NH_2_ group of first lysine (K) amino acid of lysozyme.

The spin-labeled proteins were mixed with the unlabeled ones to reduce spin concentration. The nitroxide TEMPOL was simply added to the hydrated casein. The resulting spin label concentration was measured using CW EPR, by comparison with a standard sample. In all cases, nitroxide/protein molar ratio was between 1/200 and 1/100.

The dehydrated and hydrated proteins were prepared in the following way: The obtained protein samples were dispersed on a glass support and dried over NaOH up to the state of a solid film. These films were then milled into powders and further dried for 24 h. The resulting powders were considered as dehydrated proteins. To obtain their hydrated states, these powders were stored over the water surface up their weight increase by 40%. In some cases, hydration was performed by deuterium water, D_2_O.

Measurements were carried out on an X-band ELEXSYS E580 9-GHz FT-EPR spectrometer (Bruker, Rheinstetten, Germany) equipped with a dielectric cavity (Bruker ER 4118X-MD5) inside an Oxford Instruments (Abingdon, UK) CF 935 cryostat. The cryostat was cooled by a cold nitrogen gas stream, with an accuracy of ±0.5 K. In continuous wave (CW) EPR measurements, over-modulation and saturation effects were avoided by diminishing the modulation amplitude and the microwave power to the levels at which EPR spectra become independent on these operating parameters.

In ESE measurements, the cavity was over-coupled to provide a quality *Q* near 100 and the dead time less than 120 ns. A two-pulse microwave sequence was used (90°-τ-180°-τ-echo), with the pulse length durations of 16 ns for the 90°-pulse and 32 ns for the 180°-pulse. The time interval τ was scanned starting from the initial delay of 120 ns with a step of 8 ns. To obtain echo-detected EPR spectra, the time delay τ was fixed and the magnetic field scanned. To observe the ESEEM effects, a three-pulse sequence (90°-τ-90°-T-90°-τ-echo) was employed. The time delay τ was fixed at 204 ns, and the time delay T was scanned. To eliminate unwanted echoes, a four-step phase-cycling program was used.

## 5. Conclusions

The results of this investigation show that at cryogenic temperatures the IDP casein possesses more internal freedom for molecular motions than the structured globular protein lysozyme (taken here as a reference): in the former case the stochastic molecular librations manifest themselves in ESE decays already above 100 K while in the latter one these motions appear only above 130 K. However, in casein near 120 K a kink in the temperature dependence appears—the motions with temperature increase develop slower above 120 K; and this effect is not observed for lysozyme. This slowing in casein may be explained by repacking of its molecules with temperature increase, resulting in a more cohesive and/or more rigid intermolecular structure.

The close similarity was found for motions in these two proteins with those known for a much simpler molecular system of gel-phase lipid bilayers. This analogy may imply that molecular packing in globular protein lysozyme resembles that known for the bilayer composed of conformationally ordered lipids while in IDP casein molecular packing resembles that known for the bilayer composed of conformationally disordered lipids. For the latter case, the disorder–order transition with temperature increase may be explained by the possibility for the lipid molecules to rearrange their residues in way which makes the intermolecular structure more ordered. Then one may suggest that the found temperature-induced transition at 120 K in casein is also determined by rearrangement of the peptide residues in a more ordered way.

## Supplementary Materials

Figure S1. Three-pulse stimulated ESEEM time traces for spin-labeled lysozyme and casein. Time delay τ between first two pulses is 204 ns. Temperature is 78 K, Figure S2. Echo-detected EPR spectra for hydrated (*h* = 0.4) IASL-lysozyme (a) and IASL-casein (b) taken at fixed delay τ = 120 ns at different temperatures. Spectra are normalized by their maxima, Figure S3. Left: An example of the original echo decays, E1(2τ) and E2(2τ), taken at two field positions shown in the insert, at 95 K (semi-logarithmic plot). Right: Semi-logarithmic plot for the ratios E2(2τ)/E1(2τ) for different temperatures, with the approximating straight lines, Figure S4. CW EPR spectra at different temperatures for (a) hydrated lysozyme; (b) hydrated casein. The vertical dashed lines match the positions of the extreme peaks at 120 K, Figure S5. Temperature dependences of the $\langle A_{\left|\right|}\rangle$ value found from the splitting between the external peaks in the CW EPR spectra for hydrated casein and lysozyme (see Figure S4). Dashed lines show low-temperature linear approximations.

## Sample Availability

Samples of the compounds are not available from the authors.
