# Supplementary material for: Evidence for an Ordering Transition near 120 K in an Intrinsically Disordered Protein, Casein
Source: Molecules. 2021 Oct 1;26(19):5971. doi: 10.3390/molecules26195971 (PMC8512290; doi:10.3390/molecules26195971)
Supplement: Supplementary file 1 [file molecules-26-05971-s001.zip › molecules-1375245-supplementary.pdf]

molecules

Supplementary Materials

Evidence for an Ordering Transition Near 120 K in an Intrinsically Disordered Protein, Casein

Natalya A. Maslennikova,[1,2] Elena A. Golysheva,[2] Sergei A. Dzuba[2,*]

[1] Department of Physics, Novosibirsk State University, Novosibirsk,630090, Russia; Natalya.A.Maslennikova@gmail.com
[2] Voevodsky Institute of Chemical Kinetics and Combustion, Russian Academy of Sciences, Novosibirsk,630090, Russia; elenabiochem@gmail.com
* Correspondence: dzuba@kinetics.nsc.ru

Content

(1) ESEEM Upon D_2O Hydration …………………………………...1
(2) Echo-detected EPR Spectra at Different Temperatures…..……2
(3) ESE Decays……………………………………………..………..2
(4) CW EPR Spectra at Different Temperatures……………….....3

1. ESEEM Upon D_2O Hydration

Information on the spin label location can be obtained in three-pulse stimulated ESEEM experiments in D_2O-hydrated proteins. Figure S1 shows the results of these measurements where the original ESEEM data were treated as described in [39-41]. Oscillations seen here (i.e. ESEEM) are induced by hyperfine interaction of the electron spin with deuterium nuclei.

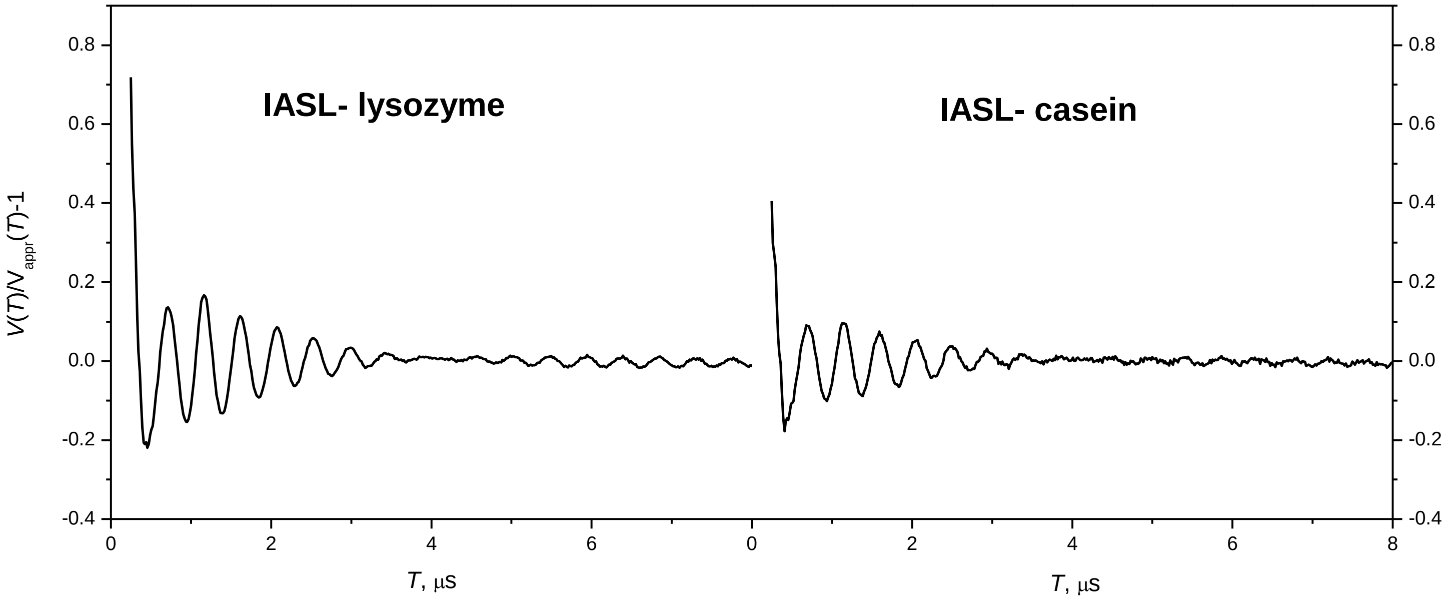

Figure S1. Three-pulse stimulated ESEEM time traces for spin-labeled lysozyme and casein. Time delay τ between first two pulses is 204 ns. Temperature is 78 K.

The amplitude of these oscillations reflects the distance of the spin label to the hydration layer. For IASL-lysozyme, this amplitude is close to that found in phospholipid bilayer [40] for spin label located on the bilayer surface, directly exposing to water (see Figure 1 in Ref. 40). For IASL-casein, the amplitude is somewhat smaller but nevertheless closer to that found in phospholipid bilayer [40] for spin label located on the bilayer

surface than to those in the bilayer interior (cf. data in Figure 1 in Ref. 40). So, in both IASL-lysozyme and IASL-casein spin labels are located at the peptide surface.

2. Echo-detected EPR Spectra at Different Temperatures

The effects of anisotropic relaxation strongly depend on temperature which is demonstrated in Figure S2 for both lysozyme and casein. Here, echo-detected EPR spectra are shown for fixed τ = 120 ns and for different temperatures.

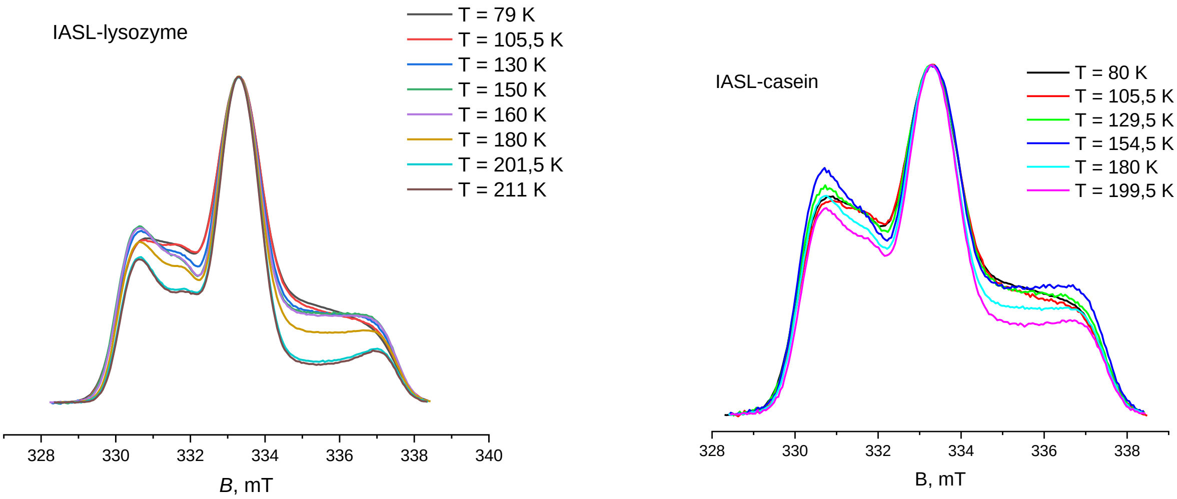

(a) **(b)**

Figure S2. Echo-detected EPR spectra for hydrated (h=0.4) IASL-lysozyme (**a**) and IASL-casein (**b**) taken at fixed delay τ = 120 ns at different temperatures. Spectra are normalized by their maxima.

One can see that in the middle of the high-field component relaxation is the fastest which is in agreement with the librational model.

3. ESE Decays

The ratio of two ESE decays obtained at two field positions with different spectral anisotropy is expected to present a pure contribution of stochastic molecular librations, and within this model may be expressed as (Equation (1)):

$$\frac{E_2(2\tau)}{E_1(2\tau)} = const\ \exp\left(-2\tau\Delta W\right) \quad (1)$$

Examples of the two original decays, $E_1(2\tau)$ and $E_2(2\tau)$, , plotted in a semilogarithmic scale, are shown in Figure S3 (left). The ratios of the decays, $E_2(2\tau)/E_1(2\tau)$, are shown in Figure S3 (right). One can see that these ratios can be approximated fairly well by straight lines which is in agreement with Equation (1).

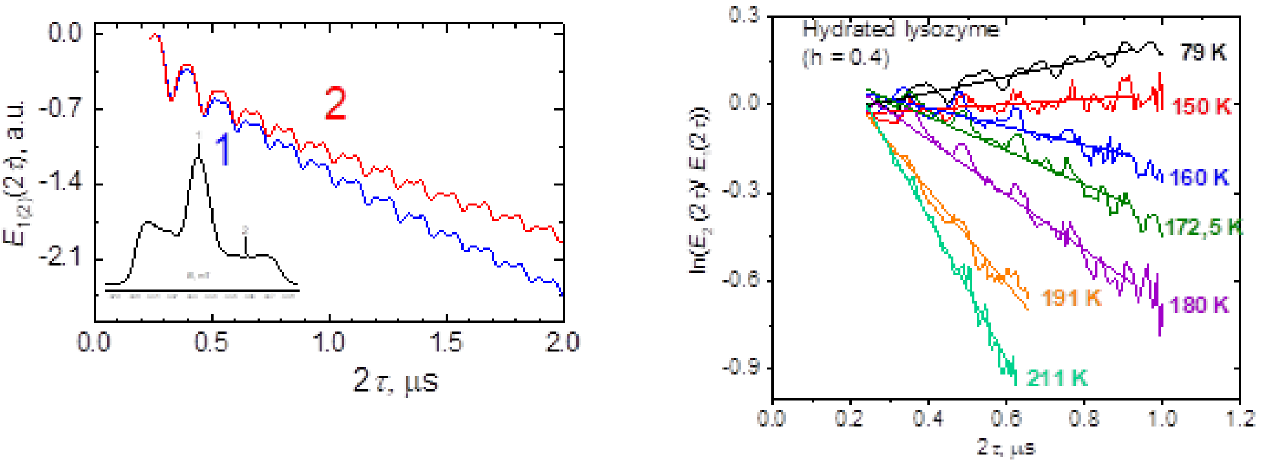

Figure S3. Left: An example of the original echo decays, $E_1(2\tau)$ and $E_2(2\tau)$, taken at two field positions shown in the insert, at 95 K (semi-logarithmic plot). Right: Semi-logarithmic plot for the ratios $E_2(2\tau)/E_1(2\tau)$ for different temperatures, with the approximating straight lines.

Figure S3 (right) shows that at low temperatures the slope is positive. This is a consequence of another field-dependent relaxation process, the so-called instantaneous diffusion mechanism (see e.g. [14]) arising because of instant modulation of the echo intensity by the microwave pules in presence of magnetic dipole-dipolar interaction between electron spins. Theory predicts that this contribution is also described by the exponential dependence, similar to Eq. (1), with a rate which we denote here as ΔW_{ID}. Then, Figure S3 (right) shows that with temperature increase, the slope gradually becomes more and more negative, which can be attributed to appearing the stochastic molecular librations, obeying Eq. (1). As the instantaneous diffusion contribution is temperature-independent in solids, it may be easily eliminated by subtracting decays obtained at low temperature. So, ΔW can be determined from the experimentally observed ΔW_{total} as (Equation (2))

$$\Delta W = \Delta W_{total} - \Delta W_{ID}. \tag{2}$$

A linear approximation of the ratios $E_2(2\tau)/E_1(2\tau)$ for all samples was conducted in the time range 2τ varying from 240 ns to 920 ns.

4. CW EPR Spectra at Different Temperatures

For fast stochastic librations as well as for dynamical librations (with a frequency larger than 10^9 Hz), the nitroxide magnetic tensors are partially averaged. This averaging manifests itself in CW EPR spectra by reducing the total spectral splitting [46]. Figure S4 shows CW EPR spectra at different temperatures for hydrated spin-labeled lysozyme and casein. The vertical dashed lines match the positions of the extreme peaks at 120 K, in order to visualize the change in their positions with increasing temperature. The total splitting between these two positions may be ascribed to the doubled principal value $A_{||}$ of the hyperfine structure tensor (A-tensor, axial approximation is employed), $2\langle A_{||}\rangle$, where angular brackets imply motional averaging. The found $\langle A_{||}\rangle$ values are given in Figure S5.

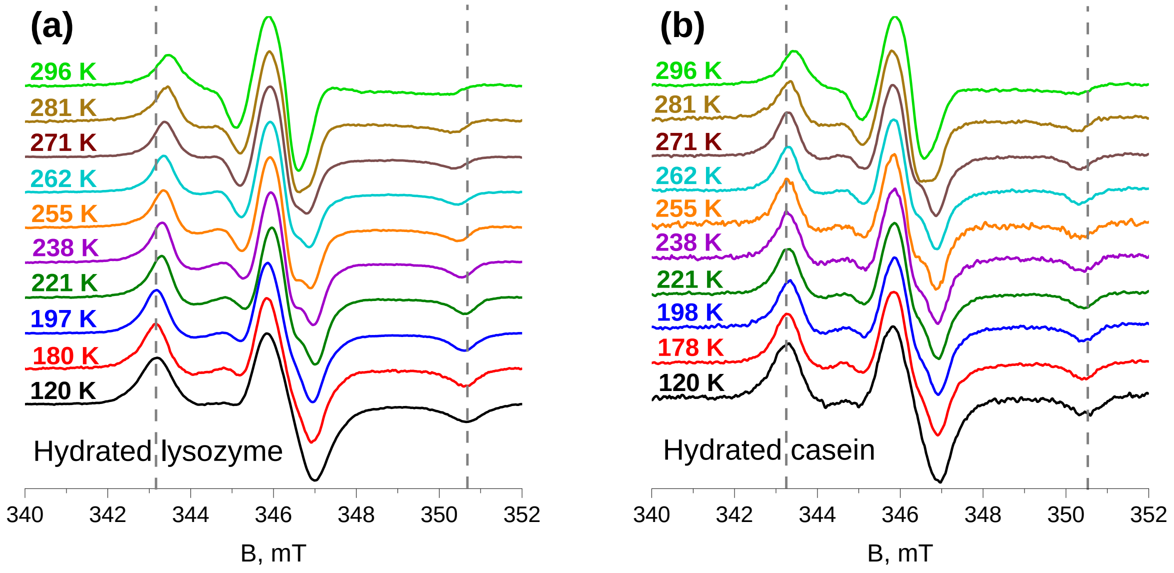

Figure S4. CW EPR spectra at different temperatures for (a) hydrated lysozyme; (b) hydrated casein. The vertical dashed lines match the positions of the extreme peaks at 120 K.

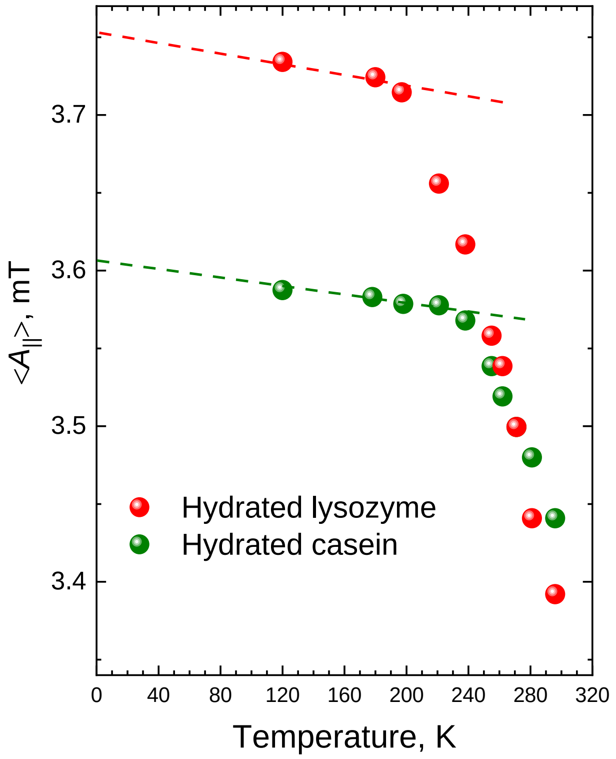

Figure S5. Temperature dependences of the $<A_{||}>$ value found from the splitting between the external peaks in the CW EPR spectra for hydrated casein and lysozyme (see Figure S4). Dashed lines show low-temperature linear approximations.
